# How long is long enough? Timing of pre-conceptional remission predicts relapse risk during pregnancy in IBD

**DOI:** 10.1093/ecco-jcc/jjaf176

**Published:** 2025-10-13

**Authors:** Dianne G Bouwknegt, Birgit Hoekstra, Hylke C Donker, Bram van Es, Henk Groen, Gerard Dijkstra, Willemijn A van Dop, Tjebbe Tauber, C Janneke van der Woude, Marijn C Visschedijk, Dutch Initiative on Crohn and Colitis (ICC), Alexander Bodelier, Lauranne Derikx, Willemijn van Dop, Marjolijn Duijvestein, Noortje Festen, Herma Fidder, Rogier Goetgebuer, Carmen Horjus, Jeroen Jansen, Bindia Jharap, Vincent de Jonge, Mark Löwenberg, Nofel Mahmmod, Sander van der Marel, Wout Mares, Peter Mensink, Andrea van der Meulen, Zlatan Mujagic, Loes Nissen, Liekele Oostenburg, Marieke Pierik, Tessa Römkens, Fiona van Schaik, Xavier Smeets, Marijn Visschedijk, Michael van der Voorn, Philip Voorneveld, Annemarie de Vries, Rachel West, Egbert-Jan van der Wouden

**Affiliations:** Department of Gastroenterology and Hepatology, University Medical Center Groningen, University of Groningen, Groningen, The Netherlands; Department of Gastroenterology and Hepatology, University Medical Center Groningen, University of Groningen, Groningen, The Netherlands; Department of Epidemiology, University Medical Center Groningen, University of Groningen, Groningen, The Netherlands; MedxAI, Amsterdam, The Netherlands; Central Diagnostic Laboratory, University Medical Center Utrecht, Utrecht, The Netherlands; Department of Epidemiology, University Medical Center Groningen, University of Groningen, Groningen, The Netherlands; Department of Gastroenterology and Hepatology, University Medical Center Groningen, University of Groningen, Groningen, The Netherlands; Department of Gastroenterology and Hepatology, Radboud University Medical Center, Nijmegen, The Netherlands; MedxAI, Amsterdam, The Netherlands; Department of Gastroenterology and Hepatology, Radboud University Medical Center, Nijmegen, The Netherlands; Department of Gastroenterology and Hepatology, Erasmus University Medical Center, Rotterdam, The Netherlands; Department of Gastroenterology and Hepatology, University Medical Center Groningen, University of Groningen, Groningen, The Netherlands

**Keywords:** inflammatory bowel disease, pregnancy, relapse, pre-conception care

## Abstract

**Background and Aims:**

Inflammatory bowel disease (IBD) often coincides with pregnancy, and disease activity during pregnancy increases the risk of adverse outcomes. We aimed to determine how disease course before conception influences relapse risk during pregnancy, adjusting for established risk factors.

**Methods:**

In this multicenter, retrospective cohort study, we included adult women with IBD who were pregnant during treatment at one of three university hospitals between 2017 and 2022. Using generalized estimating equations, we evaluated associations between relapse during pregnancy and pre-conceptional flares, categorized into three time intervals. Analyses were adjusted for phenotype, disease duration, surgical history, biologic use, smoking, and assisted reproduction. Interaction analyses were conducted with matched non-pregnant women.

**Results:**

We included 386 women (63.4% Crohn’s disease, 36.6% ulcerative colitis) with 476 pregnancies. Pre-conceptional flares were significantly associated with relapse if they occurred <3 months [adjusted odds ratio (aOR) 5.289, 95% CI 2.6-10.8, *P* < .001] or 3-6 months prior to conception (aOR 2.910, 95% CI 1.0-8.2, *P* = .043), but not 6-12 months prior (aOR 1.636, 95% CI 0.8-3.2, *P* = .146). Other predictors were not significantly associated with relapse. There was no significant interaction between pregnancy and pre-conceptional disease activity.

**Conclusions:**

This large multicenter study demonstrates that disease activity within 6 months before conception significantly increases the risk of relapse during pregnancy in women with IBD. Our study is the first to assess both the pre-conceptional disease course and a broad set of known risk factors in a real-world cohort.

## 1. Introduction

Inflammatory bowel disease (IBD) is frequently diagnosed in young adults in their reproductive years, with the peak age of diagnosis before 35 years.^1^ Many female patients will become pregnant after diagnosis. The disease and its treatment therefore often coincide with family planning and pregnancy. This unique population has been the subject of extensive research focusing on pregnancy outcomes.^2-5^ Overall, there are slightly higher rates of prematurity, low birth weight (LBW), and being small for gestational age (SGA) in children born to mothers with IBD.^2^^,^^3^ Meta-analyses show that this raised rate may be driven mainly by disease activity during pregnancy, which increases the risk of all aforementioned adverse outcomes.^4^^,^^5^

The mechanisms underlying these adverse pregnancy outcomes are multifactorial. Active disease has been associated with impaired maternal weight gain, as exacerbations of IBD often result in malabsorption, abdominal pain, and weight loss, thereby limiting the metabolic resources necessary to sustain a healthy pregnancy to term.^6^ Furthermore, vitamin D deficiency has been linked both to disease activity in IBD and adverse pregnancy outcomes.^7^^,^^8^ Additionally, dysbiosis of the gut microbiota may play a role; compromised mucosal barrier integrity during active disease could facilitate bacterial translocation to the placenta, potentially contributing to adverse pregnancy outcomes.^9^

Justifiably, adequate disease control is an important treatment goal during pregnancy.^10^^,^^11^ Previous studies have reported on risk factors for disease activity during pregnancy. Pedersen *et al.* identified longer disease duration in Crohn’s disease (CD) and the use of immunosuppressive therapy as risk factors. Further, they found that pregnant women with ulcerative colitis (UC) were more likely to experience a flare compared to their non-pregnant counterparts.^12^ In contrast, longer disease duration, the use of biologic therapy, and prior IBD-related surgery were independent protective factors against relapse during pregnancy in another analysis.^13^ A large, recent study in Denmark showed disease activity during a previous pregnancy and activity prior to conception, as well as the UC phenotype as important predictors for disease activity during pregnancy.^14^ Periconceptional disease activity has been consistently described as a risk factor for relapse during pregnancy.^15^^,^^16^

European guidelines emphasize the importance of achieving disease remission prior to pregnancy, though the precise timeframe remains undefined.^10^ The American Gastroenterological Association recommends maintaining a minimum of 3 months of steroid-free remission before conception, in accordance with the advice of a specialist Toronto consensus group meeting in 2016^11^^,^^17^ However, as of now, few studies have specifically investigated pre-conceptional flaring.^14^^,^^18^ This means that the real optimal duration of pre-conceptional disease remission required to minimize the risk of disease activity during pregnancy remains unknown. The aim of this study was to determine how the course of disease before conception influences relapse risk during pregnancy, while adjusting for previously established risk factors.

## 2. Methods

### 2.1. Study design

A retrospective cohort-study was performed in three university medical centers in the Netherlands (namely, Erasmus University Medical Center in Rotterdam, University Medical Center Groningen in Groningen, and Radboud University Medical Center in Nijmegen). Most data were extracted from electronic medical records (EMRs) automatically, using retrieval sheets. Data reported in free text could not be extracted in this manner, and were logged manually. These data included all pregnancy-outcomes and values that were missing from the automatic extraction (eg, medical history, start-date of medication). The Institutional research review board of Erasmus University Medical Center approved this study (23-12-2022, MEC-2022-0691), and waived the need for written informed consent.

### 2.2. Participants

All adult women with IBD who were treated in any of the participating hospitals between 2017 and 2022 were eligible for inclusion in this study. The objection registry was assessed, ensuring that women who objected against the re-use of medical data in scientific research were not included. We included women who were pregnant within the study period and diagnosed with IBD prior to conception. As controls, we included women who had not been pregnant (0–2 controls per pregnancy). Non-pregnant women were matched to pregnant women based on phenotype and age (within 5 years). In non-pregnant women, a dummy pregnancy period was defined corresponding to the pregnancy dates of the matched woman, and relapse risk was assessed during this timeframe.

Pregnancies ending before the 12th week of gestation were excluded, as these women may not have attended outpatient follow-up during pregnancy, making disease activity difficult to assess. Similarly, pregnancies for which follow-up took place in non-participating hospitals were excluded. Pregnancies following a (sub)total colectomy were excluded, as fecal calprotectin—a key marker used to define disease activity in this study—is not interpretable in these cases. Finally, pregnancies with periconceptional disease activity were excluded. Our rationale for this was as follows. First, the exclusion was inherent to our primary aim: evaluating how the course of disease activity before conception influences relapse risk during pregnancy. Patients are generally advised to conceive while being in remission, although the optimal remission interval remains undefined. We therefore focused specifically on women who conceived while in remission. Second, excluding pregnancies with periconceptional activity avoided the difficulty of distinguishing relapse during pregnancy from ongoing disease activity. Lastly, if no remission occurred during pregnancy in these women, they would not be able to contribute to the analysis, as no outcome could be observed.

### 2.3. Outcomes

#### 2.3.1. Descriptives

Baseline characteristics were collected at the time of conception, including age, phenotype, disease duration, Montreal classification, surgical history, medication use, and smoking status. The use of *in vitro* fertilization (IVF) and intracytoplasmic sperm injection (ICSI) was noted. Pregnancy-related diseases and pregnancy outcomes including duration of gestation, mode of delivery, birth weight, and congenital malformations were collected as well.

The following adverse pregnancy outcomes were evaluated: LBW, defined as a newborn weighing less than 2.500 g; prematurity, defined as a pregnancy lasting fewer than 37 weeks; and SGA, defined as a birth weight below the tenth percentile for the corresponding gestational age.

#### 2.3.2. Disease activity

Disease activity was defined as the occurrence of either a fecal calprotectin concentration of 200 mg/kg or higher^10^^,^^19^ and/or the prescription of budesonide or systemic corticosteroids. Pregnancies were deemed to be in remission at conception if either (1) fecal calprotectin had fallen below 200 µg/g prior to conception, or (2), based on manual review of the physician’s notes, the induction‑steroid course had been fully tapered and the same steroid was continued as a low‑dose maintenance regimen. If tapering was still ongoing at the time of conception, this was classified as part of the induction phase and therefore counted as active disease. The last pre-conceptional flare in the year prior to conception was logged and could fall in either of these three categories: within 3 months before conception, between 3 and 6 months before conception, or between 6 and 12 months before conception. Furthermore, relapse could occur at any point during pregnancy, resulting in the classification of the pregnancy as complicated by disease activity. Additionally, disease activity was recorded in the postpartum period, up to 6 months after delivery. For every pregnancy in which a corticosteroid prescription was the sole algorithmic indicator of a flare, the clinical notes were reviewed. Prescriptions clearly designated as continuation of low‑dose maintenance therapy were re‑classified as “no flare”; only documented remission‑induction courses were retained as true flare events.

### 2.4. Statistical analysis

#### 2.4.1. Descriptives

Patient and disease characteristics were summarized using frequencies and percentages for categorical variables, means with standard deviations (SD) for normally distributed continuous variables, and medians with interquartile ranges (IQR) for non-normally distributed continuous variables. Baseline differences between those who relapsed and those who remained in remission were assessed using $\chi^{2}$ and Mann–Whitney U tests.

#### 2.4.2. Risk factors for relapse during pregnancy and the postpartum period

To assess the influence of the potential risk factors while accounting for multiple pregnancies per patient, generalized estimating equation (GEE) models were performed using data from pregnant women only. Disease activity at any point during pregnancy served as the binary outcome. In this model, studyID was used as the subject variable, with pregnancy number (chronological per patient) as the within-subject variable.

First, univariable analyses were conducted for each predictor, including the most recent pre-conceptional flare (categorized as described previously), phenotype, disease duration, the presence of a surgical history, biological use, active smoking, and the use of IVF or ICSI as main effects. Pregnancies with no disease activity in the year prior served as the reference category for the pre-conceptional flare variable. Subsequently, multivariable GEE models were built to assess the adjusted influence of each predictor, with odds ratios (OR) and adjusted odds ratios (aOR), 95% confidence intervals (CI), and *P*-values reported. In addition, estimated marginal means (EMM) were calculated based on the final multivariable models to obtain model-adjusted probabilities of relapse for each category of the significant predictors.

To assess relapse risk in the postpartum period, all aforementioned predictors were included, with the addition of disease activity during pregnancy.

#### 2.4.3. Modulation of risk factors by pregnancy

To examine whether pregnancy modulates the impact of these risk factors, additional GEE analyses were performed in the full dataset of pregnant women and the matched non-pregnant women. First, univariable and multivariable analyses were performed in pregnant women and non-pregnant women separately. In the multivariable model including both groups, IVF/ICSI was excluded as it is not applicable to non-pregnant women. Predictors that demonstrated a statistically significant association with relapse in either the pregnant or non-pregnant group were incorporated into interaction analyses.

In these models, the group identifier (pregnant women with the matched non-pregnant women) was used as the subject variable to account for clustering within matched sets, and the individual participant ID within each group was specified as the within-subject variable. Each univariable interaction model included the main effects of pregnancy status and the predictor of interest, along with an interaction term, to assess whether the association between the predictor and relapse differed by pregnancy status. In multivariable interaction models, interaction terms were evaluated while adjusting for the main effects of other significant predictors. *P*-values for the interaction terms were reported to evaluate the presence of effect modification.

#### 2.4.4. Model specifications

An exchangeable correlation structure was applied in all models. All *P*-values of ≤.05 were considered statistically significant. Data analyses were conducted using IBM SPSS Statistics (v.28.0). The full syntax used for these analyses is provided in Supplementary data 1.

## 3. Results

### 3.1. Population

**Figure 1.**
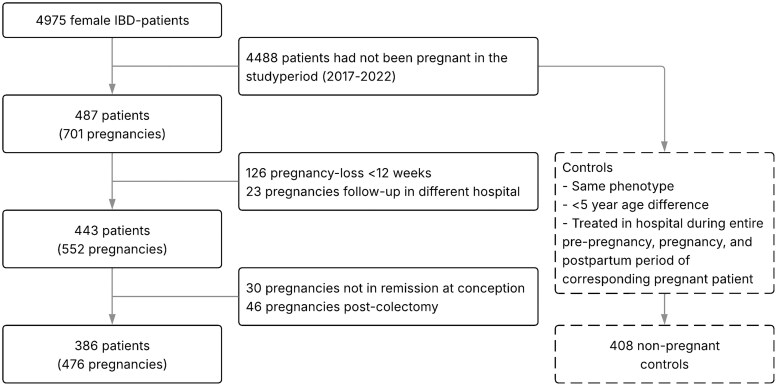
Flowchart of the inclusion of patients.

The original dataset included 4975 women, with a mean age of 43.6 years (SD 16.1) in 2017, with ages ranging from 18 to 101 years. Of these women, 487 had been pregnant in the study period, resulting in 701 pregnancies. Of these, 126 miscarriages and 23 pregnancies with a follow-up in a different hospital were excluded. Further, there were 30 pregnancies with active disease at conception. In total, 46 pregnancies were preceded by a colectomy. Ultimately, 386 patients with 476 pregnancies were included for analysis. Of the 4488 women who had not been pregnant during the study period, 408 were selected who matched the phenotype and were within 5 years of age of a pregnant patient, and who had been treated at a participating hospital throughout the pre-, mid-, and post-pregnancy periods of the matched patient. An overview of patient inclusion and exclusion is presented in Figure 1.

In 302 pregnancies (63.4%), the mother had CD, and in 174 pregnancies (36.6%), the mother had UC. Pre-conceptional disease activity was observed in 41 cases (8.6%) within 3 months prior to conception, in 22 cases (4.6%) between 3 and 6 months, and in 51 cases (10.7%) between 6 and 12 months. In the remaining 362 cases (76.1%), remission had been sustained for at least 12 months prior to conception. A flare occurred in 143 pregnancies (30.0%).

In women who remained in remission during pregnancy, UC was more often classified as proctitis (36.0% vs 16.3%, *P* = .007), whereas extensive disease was more common among those who experienced a relapse during pregnancy (46.9% vs 28.0%, *P* = .029). Those who experienced a flare during pregnancy more often used medication at conception (81.8% vs 71.2%, *P* = .015). The use of corticosteroids (3.5% vs 0.6%, *P* = .016), budesonide (3.5% vs 0.3%, *P* = .004), ustekinumab (10.5% vs 2.1%, *P* < .001), and calcineurin inhibitors (2.1% vs 0.0%, *P* = .008) as maintenance therapy was more prevalent in women with relapses during pregnancy. Lastly, these women were relatively younger at conception (30.0 vs 31.9 years, *P* < .001). Additional characteristics can be found in Table 1.

**Table 1.** Patient characteristics, pregnant women only.

	All pregnancies (*N *= 476)	Flare during pregnancy (*N *= 143)	Remission during pregnancy (*N *= 333)	*P*-value
**Phenotype (CD), *n* (%)**	302 (63.4)	94 (65.7)	208 (62.5)	.497
**Montreal classification, UC, *n* (%)**				
** Proctitis**	53 (30.5)	8 (16.3)	45 (36.0)	**.007**
** Left-sided**	44 (25.3)	14 (28.6)	30 (27.3)	.608
** Extensive**	59 (33.9)	23 (46.9)	36 (28.0)	**.029**
**Montreal classification, CD, *n* (%)**				
** Ileum**	112 (37.1)	31 (33.0)	81 (38.9)	.122
** Colon**	43 (14.2)	15 (16.0)	28 (13.5)	.772
** Ileocolic**	120 (39.7)	45 (47.9)	75 (36.1)	.160
** Upper GI**	25 (8.3)	4 (4.3)	21 (10.1)	.088
** Perianal**	46 (15.2)	18 (19.1)	28 (13.5)	.203
** Inflammatory**	196 (64.9)	68 (72.3)	128 (61.5)	.575
** Stenosing**	48 (15.9)	15 (16.0)	33 (17.3)	.692
** Fistulating**	26 (8.6)	8 (8.5)	18 (8.7)	.739
**Medication-use at conception, *n* (%)**	354 (74.4)	117 (81.8)	237 (71.2)	.015
** Amino salicylates**	115 (24.2)	39 (27.3)	76 (22.8)	.298
** Thiopurines**	131 (27.5)	43 (30.1)	88 (26.4)	.415
** Corticosteroids**	7 (1.5)	5 (3.5)	2 (0.6)	**.016**
** Budesonide**	6 (1.3)	5 (3.5)	1 (0.3)	**.004**
** Anti-TNF**	147 (30.9)	40 (28.0)	107 (32.1)	.368
** Vedolizumab**	26 (5.5)	6 (4.2)	20 (6.0)	.426
** Ustekinumab**	22 (4.6)	15 (10.5)	7 (2.1)	**<.001**
** Certolizumab**	5 (1.1)	2 (1.4)	3 (0.9)	.625
** Calcineurin inhibitors**	3 (0.6)	3 (2.1)	0 (0.0)	**.008**
**History of surgery, *n* (%)**	93 (19.5)	33 (23.1)	60 (18.0)	.202
**BMI at conception, median (IQR)**	24.6 (22.0-28.2)	24.9 (22.3-28.8)	24.5 (21.7-27.8)	.358
**Maternal age, years, median (IQR)**	31.3 (28.0-34.2)	30.0 (27.2-33.2)	31.9 (28.9-34.8)	**<.001**
**Disease duration, median (IQR)**	6.3 (3.1-10.1)	6.8 (3.1-9.8)	6.2 (3.1-11.0)	.631
**Currently smoking, *n* (%)**	48 (10.1)	16 (11.2)	32 (9.6)	.600
**Primigravida, *n* (%)**	196 (41.2)	66 (46.2)	130 (39.0)	.148
**Pregnancy conceived with IVF/ICSI, *n* (%)**	32 (6.7)	9 (6.3)	23 (6.9)	.807
**Last pre-conceptional disease activity**				**<.001**
** None in the year prior**	362 (76.1)	85 (59.4)	277 (83.2)	
** 6-12 months prior**	51 (10.7)	20 (14.0)	31 (9.3)	
** 3-6 months prior**	22 (4.6)	12 (8.4)	10 (3.0)	
** <3 months prior**	41 (8.6)	26 (18.2)	15 (4.5)	

Bold values indicate statistically significant results (*P* < .05).

Abbreviations: BMI, body mass index; CD, Crohn’s disease; ICSI, intracytoplasmic sperm injection; IQR, interquartile range; IVF, *in vitro* fertilization; *N/n*, number; UC, ulcerative colitis.

### 3.2. Disease activity within 6 months prior to conception is associated with relapse during pregnancy

Pre-conceptional disease activity was significantly associated with relapse during pregnancy (Table 2). Compared to pregnancies without disease activity in the preceding year, those with a flare within 3 months before conception had the highest risk of relapse (aOR: 5.289, 95% CI: 2.6-10.8, *P* < .001). Flares occurring 3-6 months prior were also associated with an increased relapse risk (aOR: 2.910, 95% CI: 1.0-8.2, *P* = .043), while flares 6-12 months prior were not significantly associated (aOR: 1.636, 95% CI: 0.8-3.2, *P* = .146). The EMM probability of relapse was 62% (95% CI: 41%-80%) for patients who flared within 3 months before conception, 48% (95% CI: 24%-73%) for those who flared 3-6 months prior, and 34% (95% CI: 19%-53%) for those with a flare 6-12 months prior.

**Table 2.** Associations between predictors and relapse during pregnancy.

	Univariable GEE analysis			Multivariable GEE analysis		
**Characteristic**	OR	95% CI	*P*-value	aOR	95% CI	*P*-value
**Last flare**						
** 0-3 months prior**	5.018	2.6-9.9	**<.001**	5.289	2.6-10.8	**<.001**
** 3-6 months prior**	2.897	1.1-8.0	**.039**	2.910	1.0-8.2	**.043**
** 6-12 months prior**	1.616	0.8-3.1	.152	1.636	0.8-3.2	.146
**UC**	0.934	0.6-1.4	.757	0.854	0.5-1.4	.524
**Disease duration**	0.984	1.0-1.0	.434	0.975	0.9-1.0	.204
**History of surgery**	1.264	0.8-2.1	.364	1.150	0.7-2.0	.614
**Biological use**	1.045	0.7-1.6	.836	0.904	0.6-1.4	.644
**Active smoking**	1.106	0.6-2.2	.767	1.198	0.6-2.4	.607
**IVF/ICSI pregnancy**	0.842	0.4-2.0	.698	0.762	0.3-2.0	.571

Results of the generalized estimating equations (GEE) analysis, both univariable unadjusted outcomes and multivariable adjusted outcomes, performed in pregnancies (*n *= 476) only. Bold values indicate statistically significant results (*P* < .05).

Abbreviations: aOR, adjusted odds ratio; CI, confidence interval; ICSI, intracytoplasmic sperm injection; IVF, *in vitro* fertilization; OR, odds ratio; UC, ulcerative colitis.

None of the other investigated factors—including phenotype, disease duration, surgical history, biological use, active smoking, or IVF/ICSI—were significantly associated with the risk of relapse during pregnancy in either univariable or multivariable analysis.

### 3.3. Pregnancy does not modulate the impact of predictors on relapse risk

The baseline characteristics of the non-pregnant women can be found in Supplementary data 2. In the matched non-pregnant group (*n *= 408), pre-conceptional disease activity was also significantly associated with relapse during the dummy pregnancy period. However, the association showed a less consistent gradient compared to the pregnant group: adjusted ORs were 5.632 (95% CI: 2.9-11.1, *P* < .001) for flares within 3 months prior, 3.715 (95% CI: 1.9-7.1, *P *< .001) for 3-6 months prior, and 3.269 (95% CI: 1.7-6.3, *P *< .001) for flares occurring between 6 and 12 months prior. Additionally, UC phenotype was significantly associated with a lower relapse risk in non-pregnant women (aOR: 0.536, 95% CI: 0.3-0.9, *P *= .029), whereas this association was not observed in pregnant patients. No other predictors were significantly associated with relapse.

Both pre-conceptional disease activity and IBD phenotype were evaluated in the interaction analysis. In univariable interaction models, the interaction terms between pregnancy status and pre-conceptional disease activity (*P *= .670) as well as between pregnancy status and UC phenotype (*P *= .188) were not statistically significant. In multivariable models adjusting for the relevant main effects, interaction terms again remained non-significant: *P *= .736 for disease activity (adjusted for phenotype), and *P *= .423 for phenotype (adjusted for disease ­activity). These findings suggest that pregnancy does not statistically significantly modify the association between either pre-conceptional disease activity or IBD phenotype and relapse risk. Full results of the main effect and interaction analyses are presented in Supplementary data 3.

### 3.4. Disease activity during pregnancy is a strong predictor for relapse in the postpartum period

Experiencing a flare during pregnancy was the strongest predictor of postpartum relapse (aOR: 4.477, 95% CI: 2.5-8.1, *P* < .001) (Table 3). This translated to an EMM probability of postpartum relapse of 23% (95% CI: 11%-41%) for women who experienced disease activity during pregnancy, vs a probability of 6% (95% CI: 2%-15%) for those who did not.

**Table 3.** Associations between predictors and relapse in the postpartum period.

	Univariable GEE analysis			Multivariable GEE analysis		
**Characteristic**	OR	95% CI	*P*-value	aOR	95% CI	*P*-value
**Flare during pregnancy**	5.292	3.0-9.3	**<.001**	4.477	2.5-8.1	**<.001**
**Last flare**						
** 0-3 months prior**	3.919	1.7-8.9	**.001**	2.638	1.1-6.5	**.035**
** 3-6 months prior**	3.181	1.2-8.7	**.025**	2.367	0.9-6.0	.071
** 6-12 months prior**	1.537	0.6-3.9	.371	1.481	0.6-3.5	.368
**UC**	1.237	0.7-2.2	.462	1.249	0.7-2.3	.480
**Disease duration**	1.003	1.0-1.1	.909	0.999	1.0-1.1	.985
**History of surgery**	0.813	0.4-1.9	.813	0.773	0.3-1.8	.537
**Biological use**	1.413	0.8-2.4	.214	1.379	0.8-2.5	.275
**Active smoking**	0.912	0.4-2.4	.912	0.881	0.3-2.5	.813
**IVF/ICSI pregnancy**	0.390	0.1-1.6	.194	0.354	0.1-1.3	.126

Results of the generalized estimating equation (GEE), both univariable unadjusted outcomes and multivariable adjusted outcomes, performed in pregnancies (*n *= 476) only. Bold values indicate statistically significant results (*P* < .05).

Abbreviations: CI, confidence interval; ICSI, intracytoplasmic sperm injection; IVF, *in vitro* fertilization; OR, odds ratio; UC, ulcerative colitis.

**Table 4.** Pregnancy outcomes categorized by flare-occurrence during pregnancy.

	Flare during pregnancy (*n *= 143)	Remission throughout pregnancy (*n *= 333)	*P*-value*
**Gestational week at delivery, median (IQR)**	39 (38-40)	39 (38-40)	.499
**Birth weight (g), median (IQR)**	3363 (2963-3649)	3316 (2906-3715)	.825
**Caesarean section, *n* (%)**	39 (27.3)	83 (24.9)	.591

* Mann–Whitney U test and chi-square test.

Abbreviation: IQR, interquartile range.

A history of disease activity in the preconception period also showed an association with postpartum relapse, though the effect was weaker in the adjusted model. Patients with a flare within 3 months before conception had an increased risk (aOR: 2.638, 95% CI: 1.1-6.5, *P* = .035). Flares occurring 3-6 months prior to conception (aOR: 2.367, 95% CI: 0.9-6.0, *P* = .071), and flares occurring 6-12 months before conception (aOR: 1.481, 95% CI: 0.6-3.5, *P* = .368) were not significantly associated with postpartum relapse. The EMM probability for postpartum relapse was 17% (95% CI: 5%-43%) in those with a pre-conceptional flare within 3 months prior to conception, 16% (95% CI: 6%-36%) in those with a flare 3-6 months prior, and 11% (95% CI: 3%-28%) in those with a flare 6-12 months prior. No further significant associations were found.

### 3.5. Pregnancy outcomes did not differ between those who relapsed and those who stayed in remission

In total, 57 pregnancies (12.0%) were affected by a pregnancy-related disease. These included diabetes gravidarum (*n *= 25, 5.3%), pre-eclampsia (*n *= 18, 3.8%), intrahepatic cholestasis of pregnancy (ICP) (*n *= 7, 1.5%), pregnancy-induced hypertension (*n *= 5, 1.1%), and hemolysis elevated liver enzymes and low platelets (HELLP) syndrome (*n *= 2, 0.4%).

Seven infants were born with a congenital malformation, including heart defects (*n *= 3, 0.6%), pulmonary sequestration (*n *= 2, 0.4%), trisomy 13 (*n *= 1, 0.2%), and syndactyly (*n *= 1, 0.2%).

In all pregnancies, 57 infants were born prematurely (12.0%). LBW was noted in 45 newborns (9.5%), and 58 children (12.2%) were SGA. Notably, infants born to those who experienced a flare during pregnancy and to those who did not did not differ significantly in gestational age (39 weeks vs 39 weeks, *P* = .499) or birthweight (3363 g vs 3316 g, *P* = .825) (Table 4).

## 4. Discussion

In this study, we show that pre-conceptional flaring up to 6 months prior to conception is associated with relapse during pregnancy. The shorter the interval between the last pre-conceptional flare and conception, the higher the risk of relapse during pregnancy. This increased risk for relapse after a more recent episode was comparable in those who became pregnant and those who did not; pregnancy did not modulate the magnitude of the effect of this risk factor. Experiencing a flare during pregnancy was the strongest independent predictor of postpartum relapse. Notably, in our study, pregnancy outcomes did not differ between those who had a relapse during pregnancy and those who did not. This is the first study to assess both the course of pre-conceptional disease activity across multiple intervals and established risk factors for relapse in one of the largest multicenter cohorts to date.^12–14^^,^^18^

The association between a recent flare and an increased risk of relapse may be driven by persistent subclinical disease activity. A previous flare may not have been fully resolved, leaving residual inflammation that is not detected upon routine clinical monitoring. Ongoing mucosal inflammation could predispose patients to a subsequent relapse.^20^^,^^21^ Notably, the effect size of recent disease activity as a risk factor was not influenced by pregnancy itself. While pregnancy induces immune adaptations, including a shift toward a more tolerogenic immune state and a Th2-dominant phenotype, along with increases in hormones such as HCG, estrogen, and progesterone—which have shown anti-inflammatory effects in animal models—its actual impact on IBD disease course is influenced by multiple factors. Individual patient characteristics, microbiome composition and hormone- and diet-induced changes therein, and underlying genetic risk all contribute to whether pregnancy modifies disease course.^22^

Postpartum relapse risk appears to be strongly influenced by disease activity during pregnancy. In line with previous reports,^23^ in our cohort, women who experienced active disease during pregnancy had a markedly higher risk of postpartum relapse compared to those who remained in remission. This underscores the importance of achieving and maintaining disease control throughout pregnancy, not only to optimize pregnancy outcomes but also to reduce the risk of postpartum disease activity. Pre-conceptional disease activity also contributed to postpartum relapse risk, but its effect was more modest and less consistently significant in adjusted analyses.

Interestingly, although previous studies have reported that UC patients flare more frequently during pregnancy compared to those with CD^13^^,^^14^ our analysis did not show a statistically significant difference in relapse risk by phenotype among pregnant women. In the non-pregnant group, UC patients had a significantly lower relapse risk than those with CD, but this difference was not observed during pregnancy. While the interaction between phenotype and pregnancy was not statistically significant, this trend may suggest a relative increase in relapse risk among UC patients during pregnancy. The relatively modest effect of pregnancy on relapse risk in UC, compared to earlier studies, may in part be explained by the overrepresentation of more extensive or complicated disease in the non-pregnant control group. This probably contributed to a higher baseline flare rate among controls, reducing the contrast in disease activity between pregnant and non-pregnant women and potentially underestimating the effect of pregnancy itself.

Compared to a large systematic review,^5^ the rates of adverse pregnancy outcomes in our cohort were within a similar range though modestly higher, including prematurity (12.0% vs 8.6%), LBW (9.5% vs 8.9%), and SGA newborns (12.2% vs 5.2%). A substantial proportion of women (19.5%) had a history of IBD-related surgery, which has been associated with an increased risk of adverse pregnancy outcomes, particularly prematurity, in a recent large Danish study.^24^ In addition, 10.1% of women in our cohort reported active smoking at the time of conception, which is slightly higher than the average rate of 8.1% reported for pregnant women in Europe.^25^ Given the well-established association between smoking and adverse pregnancy outcomes,^26^ this may have contributed to the moderately higher rates of adverse outcomes observed in our study.

Pregnancies ending in pregnancy-loss before the 12th week were excluded from the analyses, as disease activity data are probably unavailable at such an early stage. This may have introduced selection bias, as active disease increases the risk of pregnancy-loss. However, the observed miscarriage rate in our cohort (18%) falls within the expected range for the general population according to a recent large meta-analysis (15.3%, 95% CI: 12.5-18.7%),^27^ suggesting no major overrepresentation. Nevertheless, disease activity may have contributed in some of these cases.

Surprisingly, in our study, birth outcomes did not differ between women who experienced disease activity during pregnancy vs women who remained in remission. Recent studies from tertiary centers with specialized IBD/pregnancy clinics, conducted in the era of biologic therapy, have reported similar findings,^15^^,^^18^^,^^28^ indicating excellent pregnancy outcomes in women with IBD, even if disease activity occurred during pregnancy. We hypothesize that the stringent follow-up in these centers, combined with the current knowledge on the safety of remission-inducing drugs including biologics,^29^ may contribute to rapid disease control during pregnancy. Perhaps this proactive approach to treating flares during pregnancy helps mitigate their potential negative impact, contributing to improved pregnancy outcomes that approach those of women who remain in remission. Nevertheless, it cannot be ruled out that the relatively low number of adverse pregnancy outcomes and cases of disease activity during pregnancy in our study may have limited our ability to detect subtle differences between groups.

The main strength of our study is the large number of included pregnancies and the detailed data that were collected on all participants. Objective measures were used to assess disease activity, enhancing the reliability of our findings. To the best of our knowledge, this is the first study to examine the influence of pre-conceptional flares in different timeframes on disease activity during pregnancy in patients with IBD, while accounting for other known predictors of relapse.

A few important limitations should be considered. First, the limited number of pre‑conception flares within each defined interval resulted in wide CIs, which means that the precise magnitude of risk should be interpreted with caution. Second, due to the retrospective nature of our data collection, we could not reliably determine when a flare had resolved. As a result, indicators marking the onset of a flare may have been counted as new flares, even if they were part of the same episode. Third, calprotectin measurements may have been less frequent in women without symptoms, potentially leading to missed cases of mild flares (ie, selection bias). Relatedly, assessing disease activity at conception was challenging, as the interval between the last calprotectin measurement and conception varied. Here, we also assumed that the absence of repeat testing or induction therapy reflected remission. Future studies should therefore implement a prospective design with a clear definition of both onset and resolution of a flare, assessing disease activity at set timepoints during pregnancy.

In conclusion, pre-conceptional disease activity within 6 months prior to conception is a key risk factor for relapse during pregnancy. The increased risk for relapse after a more recent episode was comparable in those who become pregnant and those who do not; pregnancy does not modulate the magnitude of the effect of this risk factor. We recommend a 6-month disease remission period before attempting to conceive and a pro-active treatment of flares during pregnancy.

## Supplementary Material

jjaf176_Supplementary_Data

## Data Availability

The data underlying this article cannot be shared publicly in order to protect the privacy of individuals who participated in the study. The data will be shared on reasonable request to the corresponding author.
