# Supplementary material for: How long is long enough? Timing of pre-conceptional remission predicts relapse risk during pregnancy in IBD
Source: J Crohns Colitis. 2025 Oct 13;19(10):jjaf176. doi: 10.1093/ecco-jcc/jjaf176 (PMC12640223; doi:10.1093/ecco-jcc/jjaf176)
Supplement: jjaf176_Supplementary_Data [file jjaf176_supplementary_data.zip › Supplementary data 1 - Complete syntax of analyses.docx]

**Supplementary data 1**

**Complete syntax of analyses**

*Risk factors for relapse during pregnancy (performed in cases only)*

* Generalized Estimating Equations.

GENLIN Flare_during (REFERENCE=FIRST) BY Flare_last_in (ORDER=DESCENDING)

/MODEL Flare_last_in INTERCEPT=YES

DISTRIBUTION=BINOMIAL LINK=LOGIT

/CRITERIA METHOD=FISHER(1) SCALE=1 MAXITERATIONS=100 MAXSTEPHALVING=5 PCONVERGE=1E-006(ABSOLUTE)

SINGULAR=1E-012 ANALYSISTYPE=3(WALD) CILEVEL=95 LIKELIHOOD=FULL

/EMMEANS TABLES=Flare_last_in SCALE=ORIGINAL

/REPEATED SUBJECT=StudyID WITHINSUBJECT=IncludedPregnancyNr SORT=YES CORRTYPE=EXCHANGEABLE

ADJUSTCORR=YES COVB=ROBUST MAXITERATIONS=100 PCONVERGE=1e-006(ABSOLUTE) UPDATECORR=1

/MISSING CLASSMISSING=EXCLUDE

/PRINT CPS DESCRIPTIVES MODELINFO FIT SUMMARY SOLUTION (EXPONENTIATED).

* Generalized Estimating Equations.

GENLIN Flare_during (REFERENCE=FIRST) BY UC_yesno (ORDER=DESCENDING)

/MODEL UC_yesno INTERCEPT=YES

DISTRIBUTION=BINOMIAL LINK=LOGIT

/CRITERIA METHOD=FISHER(1) SCALE=1 MAXITERATIONS=100 MAXSTEPHALVING=5 PCONVERGE=1E-006(ABSOLUTE)

SINGULAR=1E-012 ANALYSISTYPE=3(WALD) CILEVEL=95 LIKELIHOOD=FULL

/EMMEANS TABLES=UC_yesno SCALE=ORIGINAL

/REPEATED SUBJECT=StudyID WITHINSUBJECT=IncludedPregnancyNr SORT=YES CORRTYPE=EXCHANGEABLE

ADJUSTCORR=YES COVB=ROBUST MAXITERATIONS=100 PCONVERGE=1e-006(ABSOLUTE) UPDATECORR=1

/MISSING CLASSMISSING=EXCLUDE

/PRINT CPS DESCRIPTIVES MODELINFO FIT SUMMARY SOLUTION (EXPONENTIATED).

* Generalized Estimating Equations.

GENLIN Flare_during (REFERENCE=FIRST) WITH DiseaseDuration

/MODEL DiseaseDuration INTERCEPT=YES

DISTRIBUTION=BINOMIAL LINK=LOGIT

/CRITERIA METHOD=FISHER(1) SCALE=1 MAXITERATIONS=100 MAXSTEPHALVING=5 PCONVERGE=1E-006(ABSOLUTE)

SINGULAR=1E-012 ANALYSISTYPE=3(WALD) CILEVEL=95 LIKELIHOOD=FULL

/REPEATED SUBJECT=StudyID WITHINSUBJECT=IncludedPregnancyNr SORT=YES CORRTYPE=EXCHANGEABLE

ADJUSTCORR=YES COVB=ROBUST MAXITERATIONS=100 PCONVERGE=1e-006(ABSOLUTE) UPDATECORR=1

/MISSING CLASSMISSING=EXCLUDE

/PRINT CPS DESCRIPTIVES MODELINFO FIT SUMMARY SOLUTION (EXPONENTIATED).

* Generalized Estimating Equations.

GENLIN Flare_during (REFERENCE=FIRST) BY Previous_Surgery_yesno (ORDER=DESCENDING)

/MODEL Previous_Surgery_yesno INTERCEPT=YES

DISTRIBUTION=BINOMIAL LINK=LOGIT

/CRITERIA METHOD=FISHER(1) SCALE=1 MAXITERATIONS=100 MAXSTEPHALVING=5 PCONVERGE=1E-006(ABSOLUTE)

SINGULAR=1E-012 ANALYSISTYPE=3(WALD) CILEVEL=95 LIKELIHOOD=FULL

/EMMEANS TABLES=Previous_Surgery_yesno SCALE=ORIGINAL

/REPEATED SUBJECT=StudyID WITHINSUBJECT=IncludedPregnancyNr SORT=YES CORRTYPE=EXCHANGEABLE

ADJUSTCORR=YES COVB=ROBUST MAXITERATIONS=100 PCONVERGE=1e-006(ABSOLUTE) UPDATECORR=1

/MISSING CLASSMISSING=EXCLUDE

/PRINT CPS DESCRIPTIVES MODELINFO FIT SUMMARY SOLUTION (EXPONENTIATED).

* Generalized Estimating Equations.

GENLIN Flare_during (REFERENCE=FIRST) BY Med_Biological_yesno (ORDER=DESCENDING)

/MODEL Med_Biological_yesno INTERCEPT=YES

DISTRIBUTION=BINOMIAL LINK=LOGIT

/CRITERIA METHOD=FISHER(1) SCALE=1 MAXITERATIONS=100 MAXSTEPHALVING=5 PCONVERGE=1E-006(ABSOLUTE)

SINGULAR=1E-012 ANALYSISTYPE=3(WALD) CILEVEL=95 LIKELIHOOD=FULL

/EMMEANS TABLES=Med_Biological_yesno SCALE=ORIGINAL

/REPEATED SUBJECT=StudyID WITHINSUBJECT=IncludedPregnancyNr SORT=YES CORRTYPE=EXCHANGEABLE

ADJUSTCORR=YES COVB=ROBUST MAXITERATIONS=100 PCONVERGE=1e-006(ABSOLUTE) UPDATECORR=1

/MISSING CLASSMISSING=EXCLUDE

/PRINT CPS DESCRIPTIVES MODELINFO FIT SUMMARY SOLUTION (EXPONENTIATED).

* Generalized Estimating Equations.

GENLIN Flare_during (REFERENCE=FIRST) BY Smoking_current (ORDER=DESCENDING)

/MODEL Smoking_current INTERCEPT=YES

DISTRIBUTION=BINOMIAL LINK=LOGIT

/CRITERIA METHOD=FISHER(1) SCALE=1 MAXITERATIONS=100 MAXSTEPHALVING=5 PCONVERGE=1E-006(ABSOLUTE)

SINGULAR=1E-012 ANALYSISTYPE=3(WALD) CILEVEL=95 LIKELIHOOD=FULL

/EMMEANS TABLES=Smoking_current SCALE=ORIGINAL

/REPEATED SUBJECT=StudyID WITHINSUBJECT=IncludedPregnancyNr SORT=YES CORRTYPE=EXCHANGEABLE

ADJUSTCORR=YES COVB=ROBUST MAXITERATIONS=100 PCONVERGE=1e-006(ABSOLUTE) UPDATECORR=1

/MISSING CLASSMISSING=EXCLUDE

/PRINT CPS DESCRIPTIVES MODELINFO FIT SUMMARY SOLUTION (EXPONENTIATED).

* Generalized Estimating Equations.

GENLIN Flare_during (REFERENCE=FIRST) BY Pregnancy_IVF_ICSI_yesno (ORDER=DESCENDING)

/MODEL Pregnancy_IVF_ICSI_yesno INTERCEPT=YES

DISTRIBUTION=BINOMIAL LINK=LOGIT

/CRITERIA METHOD=FISHER(1) SCALE=1 MAXITERATIONS=100 MAXSTEPHALVING=5 PCONVERGE=1E-006(ABSOLUTE)

SINGULAR=1E-012 ANALYSISTYPE=3(WALD) CILEVEL=95 LIKELIHOOD=FULL

/EMMEANS TABLES=Pregnancy_IVF_ICSI_yesno SCALE=ORIGINAL

/REPEATED SUBJECT=StudyID WITHINSUBJECT=IncludedPregnancyNr SORT=YES CORRTYPE=EXCHANGEABLE

ADJUSTCORR=YES COVB=ROBUST MAXITERATIONS=100 PCONVERGE=1e-006(ABSOLUTE) UPDATECORR=1

/MISSING CLASSMISSING=EXCLUDE

/PRINT CPS DESCRIPTIVES MODELINFO FIT SUMMARY SOLUTION (EXPONENTIATED).

* Generalized Estimating Equations.

GENLIN Flare_during (REFERENCE=FIRST) BY Flare_last_in UC_yesno Previous_Surgery_yesno

Med_Biological_yesno Smoking_current Pregnancy_IVF_ICSI_yesno (ORDER=DESCENDING) WITH

DiseaseDuration

/MODEL Flare_last_in UC_yesno DiseaseDuration Previous_Surgery_yesno Med_Biological_yesno

Smoking_current Pregnancy_IVF_ICSI_yesno INTERCEPT=YES

DISTRIBUTION=BINOMIAL LINK=LOGIT

/CRITERIA METHOD=FISHER(1) SCALE=1 MAXITERATIONS=100 MAXSTEPHALVING=5 PCONVERGE=1E-006(ABSOLUTE)

SINGULAR=1E-012 ANALYSISTYPE=3(WALD) CILEVEL=95 LIKELIHOOD=FULL

/EMMEANS TABLES=Flare_last_in SCALE=ORIGINAL

/EMMEANS TABLES=UC_yesno SCALE=ORIGINAL

/EMMEANS TABLES=Previous_Surgery_yesno SCALE=ORIGINAL

/EMMEANS TABLES=Med_Biological_yesno SCALE=ORIGINAL

/EMMEANS TABLES=Smoking_current SCALE=ORIGINAL

/EMMEANS TABLES=Pregnancy_IVF_ICSI_yesno SCALE=ORIGINAL

/REPEATED SUBJECT=StudyID WITHINSUBJECT=IncludedPregnancyNr SORT=YES CORRTYPE=EXCHANGEABLE

ADJUSTCORR=YES COVB=ROBUST MAXITERATIONS=100 PCONVERGE=1e-006(ABSOLUTE) UPDATECORR=1

/MISSING CLASSMISSING=EXCLUDE

/PRINT CPS DESCRIPTIVES MODELINFO FIT SUMMARY SOLUTION (EXPONENTIATED).

*Modulation of risk factors by pregnancy: analysis on main effects in cases*

Main effects analyses stratified for cases and controls. For cases, only the multivariable analysis is different from the analysis above as it does not include IVF/ICSI. The multivariable analysis is therefore repeated.

* Generalized Estimating Equations.

GENLIN Flare_during (REFERENCE=FIRST) BY Flare_last_in UC_yesno Previous_Surgery_yesno

Med_Biological_yesno Smoking_current (ORDER=DESCENDING) WITH

DiseaseDuration

/MODEL Flare_last_in UC_yesno DiseaseDuration Previous_Surgery_yesno Med_Biological_yesno

Smoking_current INTERCEPT=YES

DISTRIBUTION=BINOMIAL LINK=LOGIT

/CRITERIA METHOD=FISHER(1) SCALE=1 MAXITERATIONS=100 MAXSTEPHALVING=5 PCONVERGE=1E-006(ABSOLUTE)

SINGULAR=1E-012 ANALYSISTYPE=3(WALD) CILEVEL=95 LIKELIHOOD=FULL

/EMMEANS TABLES=Flare_last_in SCALE=ORIGINAL

/EMMEANS TABLES=UC_yesno SCALE=ORIGINAL

/EMMEANS TABLES=Previous_Surgery_yesno SCALE=ORIGINAL

/EMMEANS TABLES=Med_Biological_yesno SCALE=ORIGINAL

/EMMEANS TABLES=Smoking_current SCALE=ORIGINAL

/REPEATED SUBJECT=StudyID WITHINSUBJECT=IncludedPregnancyNr SORT=YES CORRTYPE=EXCHANGEABLE

ADJUSTCORR=YES COVB=ROBUST MAXITERATIONS=100 PCONVERGE=1e-006(ABSOLUTE) UPDATECORR=1

/MISSING CLASSMISSING=EXCLUDE

/PRINT CPS DESCRIPTIVES MODELINFO FIT SUMMARY SOLUTION (EXPONENTIATED).

*Modulation of risk factors by pregnancy: analysis on main effects in controls*

All univariable and multivariable analyses are repeated in the non-pregnant controls.

* Generalized Estimating Equations.

GENLIN Flare_during (REFERENCE=FIRST) BY Flare_last_in (ORDER=DESCENDING)

/MODEL Flare_last_in INTERCEPT=YES

DISTRIBUTION=BINOMIAL LINK=LOGIT

/CRITERIA METHOD=FISHER(1) SCALE=1 MAXITERATIONS=100 MAXSTEPHALVING=5 PCONVERGE=1E-006(ABSOLUTE)

SINGULAR=1E-012 ANALYSISTYPE=3(WALD) CILEVEL=95 LIKELIHOOD=FULL

/EMMEANS TABLES=Flare_last_in SCALE=ORIGINAL

/REPEATED SUBJECT=StudyID WITHINSUBJECT=IncludedPregnancyNr SORT=YES CORRTYPE=EXCHANGEABLE ADJUSTCORR=YES

COVB=ROBUST MAXITERATIONS=100 PCONVERGE=1e-006(ABSOLUTE) UPDATECORR=1

/MISSING CLASSMISSING=EXCLUDE

/PRINT CPS DESCRIPTIVES MODELINFO FIT SUMMARY SOLUTION (EXPONENTIATED).

* Generalized Estimating Equations.

GENLIN Flare_during (REFERENCE=FIRST) BY UC_yesno (ORDER=DESCENDING)

/MODEL UC_yesno INTERCEPT=YES

DISTRIBUTION=BINOMIAL LINK=LOGIT

/CRITERIA METHOD=FISHER(1) SCALE=1 MAXITERATIONS=100 MAXSTEPHALVING=5 PCONVERGE=1E-006(ABSOLUTE)

SINGULAR=1E-012 ANALYSISTYPE=3(WALD) CILEVEL=95 LIKELIHOOD=FULL

/EMMEANS TABLES=UC_yesno SCALE=ORIGINAL

/REPEATED SUBJECT=StudyID WITHINSUBJECT=IncludedPregnancyNr SORT=YES CORRTYPE=EXCHANGEABLE ADJUSTCORR=YES

COVB=ROBUST MAXITERATIONS=100 PCONVERGE=1e-006(ABSOLUTE) UPDATECORR=1

/MISSING CLASSMISSING=EXCLUDE

/PRINT CPS DESCRIPTIVES MODELINFO FIT SUMMARY SOLUTION (EXPONENTIATED).

* Generalized Estimating Equations.

GENLIN Flare_during (REFERENCE=FIRST) WITH DiseaseDuration

/MODEL DiseaseDuration INTERCEPT=YES

DISTRIBUTION=BINOMIAL LINK=LOGIT

/CRITERIA METHOD=FISHER(1) SCALE=1 MAXITERATIONS=100 MAXSTEPHALVING=5 PCONVERGE=1E-006(ABSOLUTE)

SINGULAR=1E-012 ANALYSISTYPE=3(WALD) CILEVEL=95 LIKELIHOOD=FULL

/REPEATED SUBJECT=StudyID WITHINSUBJECT=IncludedPregnancyNr SORT=YES CORRTYPE=EXCHANGEABLE ADJUSTCORR=YES

COVB=ROBUST MAXITERATIONS=100 PCONVERGE=1e-006(ABSOLUTE) UPDATECORR=1

/MISSING CLASSMISSING=EXCLUDE

/PRINT CPS DESCRIPTIVES MODELINFO FIT SUMMARY SOLUTION (EXPONENTIATED).

* Generalized Estimating Equations.

GENLIN Flare_during (REFERENCE=FIRST) BY Previous_Surgery_yesno (ORDER=DESCENDING)

/MODEL Previous_Surgery_yesno INTERCEPT=YES

DISTRIBUTION=BINOMIAL LINK=LOGIT

/CRITERIA METHOD=FISHER(1) SCALE=1 MAXITERATIONS=100 MAXSTEPHALVING=5 PCONVERGE=1E-006(ABSOLUTE)

SINGULAR=1E-012 ANALYSISTYPE=3(WALD) CILEVEL=95 LIKELIHOOD=FULL

/EMMEANS TABLES=Previous_Surgery_yesno SCALE=ORIGINAL

/REPEATED SUBJECT=StudyID WITHINSUBJECT=IncludedPregnancyNr SORT=YES CORRTYPE=EXCHANGEABLE ADJUSTCORR=YES

COVB=ROBUST MAXITERATIONS=100 PCONVERGE=1e-006(ABSOLUTE) UPDATECORR=1

/MISSING CLASSMISSING=EXCLUDE

/PRINT CPS DESCRIPTIVES MODELINFO FIT SUMMARY SOLUTION (EXPONENTIATED).

* Generalized Estimating Equations.

GENLIN Flare_during (REFERENCE=FIRST) BY Med_Biological_yesno (ORDER=DESCENDING)

/MODEL Med_Biological_yesno INTERCEPT=YES

DISTRIBUTION=BINOMIAL LINK=LOGIT

/CRITERIA METHOD=FISHER(1) SCALE=1 MAXITERATIONS=100 MAXSTEPHALVING=5 PCONVERGE=1E-006(ABSOLUTE)

SINGULAR=1E-012 ANALYSISTYPE=3(WALD) CILEVEL=95 LIKELIHOOD=FULL

/EMMEANS TABLES=Med_Biological_yesno SCALE=ORIGINAL

/REPEATED SUBJECT=StudyID WITHINSUBJECT=IncludedPregnancyNr SORT=YES CORRTYPE=EXCHANGEABLE ADJUSTCORR=YES

COVB=ROBUST MAXITERATIONS=100 PCONVERGE=1e-006(ABSOLUTE) UPDATECORR=1

/MISSING CLASSMISSING=EXCLUDE

/PRINT CPS DESCRIPTIVES MODELINFO FIT SUMMARY SOLUTION (EXPONENTIATED).

* Generalized Estimating Equations.

GENLIN Flare_during (REFERENCE=FIRST) BY Smoking_current (ORDER=DESCENDING)

/MODEL Smoking_current INTERCEPT=YES

DISTRIBUTION=BINOMIAL LINK=LOGIT

/CRITERIA METHOD=FISHER(1) SCALE=1 MAXITERATIONS=100 MAXSTEPHALVING=5 PCONVERGE=1E-006(ABSOLUTE)

SINGULAR=1E-012 ANALYSISTYPE=3(WALD) CILEVEL=95 LIKELIHOOD=FULL

/EMMEANS TABLES=Smoking_current SCALE=ORIGINAL

/REPEATED SUBJECT=StudyID WITHINSUBJECT=IncludedPregnancyNr SORT=YES CORRTYPE=EXCHANGEABLE ADJUSTCORR=YES

COVB=ROBUST MAXITERATIONS=100 PCONVERGE=1e-006(ABSOLUTE) UPDATECORR=1

/MISSING CLASSMISSING=EXCLUDE

/PRINT CPS DESCRIPTIVES MODELINFO FIT SUMMARY SOLUTION (EXPONENTIATED).

* Generalized Estimating Equations.

GENLIN Flare_during (REFERENCE=FIRST) BY Flare_last_in UC_yesno Previous_Surgery_yesno

Med_Biological_yesno Smoking_current (ORDER=DESCENDING) WITH DiseaseDuration

/MODEL Flare_last_in UC_yesno DiseaseDuration Previous_Surgery_yesno Med_Biological_yesno

Smoking_current INTERCEPT=YES

DISTRIBUTION=BINOMIAL LINK=LOGIT

/CRITERIA METHOD=FISHER(1) SCALE=1 MAXITERATIONS=100 MAXSTEPHALVING=5 PCONVERGE=1E-006(ABSOLUTE)

SINGULAR=1E-012 ANALYSISTYPE=3(WALD) CILEVEL=95 LIKELIHOOD=FULL

/EMMEANS TABLES=Smoking_current SCALE=ORIGINAL

/EMMEANS TABLES=Flare_last_in SCALE=ORIGINAL

/EMMEANS TABLES=UC_yesno SCALE=ORIGINAL

/EMMEANS TABLES=Previous_Surgery_yesno SCALE=ORIGINAL

/EMMEANS TABLES=Med_Biological_yesno SCALE=ORIGINAL

/EMMEANS TABLES=Smoking_current SCALE=ORIGINAL

/REPEATED SUBJECT=StudyID WITHINSUBJECT=IncludedPregnancyNr SORT=YES CORRTYPE=EXCHANGEABLE ADJUSTCORR=YES

COVB=ROBUST MAXITERATIONS=100 PCONVERGE=1e-006(ABSOLUTE) UPDATECORR=1

/MISSING CLASSMISSING=EXCLUDE

/PRINT CPS DESCRIPTIVES MODELINFO FIT SUMMARY SOLUTION (EXPONENTIATED).

*Modulation of risk factors by pregnancy: analysis on the interaction between predictors and pregnancy*

Interaction analysis, performed in significant main effect variables (pre-conceptional flaring and phenotype) only. First, univariable analyses.

* Generalized Estimating Equations.

GENLIN Flare_during (REFERENCE=FIRST) BY Group Flare_last_in (ORDER=DESCENDING)

/MODEL Group Flare_last_in Group*Flare_last_in INTERCEPT=YES

DISTRIBUTION=BINOMIAL LINK=LOGIT

/CRITERIA METHOD=FISHER(1) SCALE=1 MAXITERATIONS=100 MAXSTEPHALVING=5 PCONVERGE=1E-006(ABSOLUTE)

SINGULAR=1E-012 ANALYSISTYPE=3(WALD) CILEVEL=95 LIKELIHOOD=FULL

/EMMEANS TABLES=Group SCALE=ORIGINAL

/EMMEANS TABLES=Flare_last_in SCALE=ORIGINAL

/EMMEANS TABLES=Group*Flare_last_in SCALE=ORIGINAL

/REPEATED SUBJECT=MatchedGroup WITHINSUBJECT=groupmember SORT=YES CORRTYPE=EXCHANGEABLE

ADJUSTCORR=YES COVB=ROBUST MAXITERATIONS=100 PCONVERGE=1e-006(ABSOLUTE) UPDATECORR=1

/MISSING CLASSMISSING=EXCLUDE

/PRINT CPS DESCRIPTIVES MODELINFO FIT SUMMARY SOLUTION (EXPONENTIATED).

* Generalized Estimating Equations.

GENLIN Flare_during (REFERENCE=FIRST) BY Group IBD_Subtype (ORDER=DESCENDING)

/MODEL Group IBD_Subtype Group*IBD_Subtype INTERCEPT=YES

DISTRIBUTION=BINOMIAL LINK=LOGIT

/CRITERIA METHOD=FISHER(1) SCALE=1 MAXITERATIONS=100 MAXSTEPHALVING=5 PCONVERGE=1E-006(ABSOLUTE)

SINGULAR=1E-012 ANALYSISTYPE=3(WALD) CILEVEL=95 LIKELIHOOD=FULL

/EMMEANS TABLES=Group SCALE=ORIGINAL

/EMMEANS TABLES=IBD_Subtype SCALE=ORIGINAL

/EMMEANS TABLES=Group*IBD_Subtype SCALE=ORIGINAL

/REPEATED SUBJECT=MatchedGroup WITHINSUBJECT=groupmember SORT=YES CORRTYPE=EXCHANGEABLE

ADJUSTCORR=YES COVB=ROBUST MAXITERATIONS=100 PCONVERGE=1e-006(ABSOLUTE) UPDATECORR=1

/MISSING CLASSMISSING=EXCLUDE

/PRINT CPS DESCRIPTIVES MODELINFO FIT SUMMARY SOLUTION (EXPONENTIATED).

Next, the multivariable analyses: Here, we assess the interaction term of the significant predictors (pre-conceptional flaring and phenotype), while accounting for the influence of the main effect of the other predictor.

* Generalized Estimating Equations.

GENLIN Flare_during (REFERENCE=FIRST) BY Group Flare_last_in IBD_Subtype (ORDER=DESCENDING)

/MODEL Group Flare_last_in Group*Flare_last_in IBD_Subtype INTERCEPT=YES

DISTRIBUTION=BINOMIAL LINK=LOGIT

/CRITERIA METHOD=FISHER(1) SCALE=1 MAXITERATIONS=100 MAXSTEPHALVING=5 PCONVERGE=1E-006(ABSOLUTE)

SINGULAR=1E-012 ANALYSISTYPE=3(WALD) CILEVEL=95 LIKELIHOOD=FULL

/EMMEANS TABLES=Group SCALE=ORIGINAL

/EMMEANS TABLES=Flare_last_in SCALE=ORIGINAL

/EMMEANS TABLES=Group*Flare_last_in SCALE=ORIGINAL

/EMMEANS TABLES=IBD_Subtype SCALE=ORIGINAL

/REPEATED SUBJECT=MatchedGroup WITHINSUBJECT=groupmember SORT=YES CORRTYPE=EXCHANGEABLE

ADJUSTCORR=YES COVB=ROBUST MAXITERATIONS=100 PCONVERGE=1e-006(ABSOLUTE) UPDATECORR=1

/MISSING CLASSMISSING=EXCLUDE

/PRINT CPS DESCRIPTIVES MODELINFO FIT SUMMARY SOLUTION (EXPONENTIATED).

* Generalized Estimating Equations.

GENLIN Flare_during (REFERENCE=FIRST) BY Group IBD_Subtype Flare_last_in (ORDER=DESCENDING)

/MODEL Group IBD_Subtype Group*IBD_Subtype Flare_last_in INTERCEPT=YES

DISTRIBUTION=BINOMIAL LINK=LOGIT

/CRITERIA METHOD=FISHER(1) SCALE=1 MAXITERATIONS=100 MAXSTEPHALVING=5 PCONVERGE=1E-006(ABSOLUTE)

SINGULAR=1E-012 ANALYSISTYPE=3(WALD) CILEVEL=95 LIKELIHOOD=FULL

/EMMEANS TABLES=Group SCALE=ORIGINAL

/EMMEANS TABLES=IBD_Subtype SCALE=ORIGINAL

/EMMEANS TABLES=Group*IBD_Subtype SCALE=ORIGINAL

/EMMEANS TABLES=Flare_last_in SCALE=ORIGINAL

/REPEATED SUBJECT=MatchedGroup WITHINSUBJECT=groupmember SORT=YES CORRTYPE=EXCHANGEABLE

ADJUSTCORR=YES COVB=ROBUST MAXITERATIONS=100 PCONVERGE=1e-006(ABSOLUTE) UPDATECORR=1

/MISSING CLASSMISSING=EXCLUDE

/PRINT CPS DESCRIPTIVES MODELINFO FIT SUMMARY SOLUTION (EXPONENTIATED).

*Risk factors for relapse in the postpartum period (performed in cases only)*

* Generalized Estimating Equations.

GENLIN Flare_postpartum (REFERENCE=FIRST) BY Flare_during (ORDER=DESCENDING)

/MODEL Flare_during INTERCEPT=YES

DISTRIBUTION=BINOMIAL LINK=LOGIT

/CRITERIA METHOD=FISHER(1) SCALE=1 MAXITERATIONS=100 MAXSTEPHALVING=5 PCONVERGE=1E-006(ABSOLUTE)

SINGULAR=1E-012 ANALYSISTYPE=3(WALD) CILEVEL=95 LIKELIHOOD=FULL

/EMMEANS TABLES=Flare_during SCALE=ORIGINAL

/REPEATED SUBJECT=StudyID WITHINSUBJECT=IncludedPregnancyNr SORT=YES CORRTYPE=EXCHANGEABLE

ADJUSTCORR=YES COVB=ROBUST MAXITERATIONS=100 PCONVERGE=1e-006(ABSOLUTE) UPDATECORR=1

/MISSING CLASSMISSING=EXCLUDE

/PRINT CPS DESCRIPTIVES MODELINFO FIT SUMMARY SOLUTION (EXPONENTIATED).

* Generalized Estimating Equations.

GENLIN Flare_postpartum (REFERENCE=FIRST) BY Flare_last_in (ORDER=DESCENDING)

/MODEL Flare_last_in INTERCEPT=YES

DISTRIBUTION=BINOMIAL LINK=LOGIT

/CRITERIA METHOD=FISHER(1) SCALE=1 MAXITERATIONS=100 MAXSTEPHALVING=5 PCONVERGE=1E-006(ABSOLUTE)

SINGULAR=1E-012 ANALYSISTYPE=3(WALD) CILEVEL=95 LIKELIHOOD=FULL

/EMMEANS TABLES=Flare_last_in SCALE=ORIGINAL

/REPEATED SUBJECT=StudyID WITHINSUBJECT=IncludedPregnancyNr SORT=YES CORRTYPE=EXCHANGEABLE

ADJUSTCORR=YES COVB=ROBUST MAXITERATIONS=100 PCONVERGE=1e-006(ABSOLUTE) UPDATECORR=1

/MISSING CLASSMISSING=EXCLUDE

/PRINT CPS DESCRIPTIVES MODELINFO FIT SUMMARY SOLUTION (EXPONENTIATED).

* Generalized Estimating Equations.

GENLIN Flare_postpartum (REFERENCE=FIRST) BY UC_yesno (ORDER=DESCENDING)

/MODEL UC_yesno INTERCEPT=YES

DISTRIBUTION=BINOMIAL LINK=LOGIT

/CRITERIA METHOD=FISHER(1) SCALE=1 MAXITERATIONS=100 MAXSTEPHALVING=5 PCONVERGE=1E-006(ABSOLUTE)

SINGULAR=1E-012 ANALYSISTYPE=3(WALD) CILEVEL=95 LIKELIHOOD=FULL

/EMMEANS TABLES=UC_yesno SCALE=ORIGINAL

/REPEATED SUBJECT=StudyID WITHINSUBJECT=IncludedPregnancyNr SORT=YES CORRTYPE=EXCHANGEABLE

ADJUSTCORR=YES COVB=ROBUST MAXITERATIONS=100 PCONVERGE=1e-006(ABSOLUTE) UPDATECORR=1

/MISSING CLASSMISSING=EXCLUDE

/PRINT CPS DESCRIPTIVES MODELINFO FIT SUMMARY SOLUTION (EXPONENTIATED).

* Generalized Estimating Equations.

GENLIN Flare_postpartum (REFERENCE=FIRST) WITH DiseaseDuration

/MODEL DiseaseDuration INTERCEPT=YES

DISTRIBUTION=BINOMIAL LINK=LOGIT

/CRITERIA METHOD=FISHER(1) SCALE=1 MAXITERATIONS=100 MAXSTEPHALVING=5 PCONVERGE=1E-006(ABSOLUTE)

SINGULAR=1E-012 ANALYSISTYPE=3(WALD) CILEVEL=95 LIKELIHOOD=FULL

/REPEATED SUBJECT=StudyID WITHINSUBJECT=IncludedPregnancyNr SORT=YES CORRTYPE=EXCHANGEABLE

ADJUSTCORR=YES COVB=ROBUST MAXITERATIONS=100 PCONVERGE=1e-006(ABSOLUTE) UPDATECORR=1

/MISSING CLASSMISSING=EXCLUDE

/PRINT CPS DESCRIPTIVES MODELINFO FIT SUMMARY SOLUTION (EXPONENTIATED).

* Generalized Estimating Equations.

GENLIN Flare_postpartum (REFERENCE=FIRST) BY Previous_Surgery_yesno (ORDER=DESCENDING)

/MODEL Previous_Surgery_yesno INTERCEPT=YES

DISTRIBUTION=BINOMIAL LINK=LOGIT

/CRITERIA METHOD=FISHER(1) SCALE=1 MAXITERATIONS=100 MAXSTEPHALVING=5 PCONVERGE=1E-006(ABSOLUTE)

SINGULAR=1E-012 ANALYSISTYPE=3(WALD) CILEVEL=95 LIKELIHOOD=FULL

/EMMEANS TABLES=Previous_Surgery_yesno SCALE=ORIGINAL

/REPEATED SUBJECT=StudyID WITHINSUBJECT=IncludedPregnancyNr SORT=YES CORRTYPE=EXCHANGEABLE

ADJUSTCORR=YES COVB=ROBUST MAXITERATIONS=100 PCONVERGE=1e-006(ABSOLUTE) UPDATECORR=1

/MISSING CLASSMISSING=EXCLUDE

/PRINT CPS DESCRIPTIVES MODELINFO FIT SUMMARY SOLUTION (EXPONENTIATED).

* Generalized Estimating Equations.

GENLIN Flare_postpartum (REFERENCE=FIRST) BY Med_Biological_yesno (ORDER=DESCENDING)

/MODEL Med_Biological_yesno INTERCEPT=YES

DISTRIBUTION=BINOMIAL LINK=LOGIT

/CRITERIA METHOD=FISHER(1) SCALE=1 MAXITERATIONS=100 MAXSTEPHALVING=5 PCONVERGE=1E-006(ABSOLUTE)

SINGULAR=1E-012 ANALYSISTYPE=3(WALD) CILEVEL=95 LIKELIHOOD=FULL

/EMMEANS TABLES=Med_Biological_yesno SCALE=ORIGINAL

/REPEATED SUBJECT=StudyID WITHINSUBJECT=IncludedPregnancyNr SORT=YES CORRTYPE=EXCHANGEABLE

ADJUSTCORR=YES COVB=ROBUST MAXITERATIONS=100 PCONVERGE=1e-006(ABSOLUTE) UPDATECORR=1

/MISSING CLASSMISSING=EXCLUDE

/PRINT CPS DESCRIPTIVES MODELINFO FIT SUMMARY SOLUTION (EXPONENTIATED).

* Generalized Estimating Equations.

GENLIN Flare_postpartum (REFERENCE=FIRST) BY Smoking_current (ORDER=DESCENDING)

/MODEL Smoking_current INTERCEPT=YES

DISTRIBUTION=BINOMIAL LINK=LOGIT

/CRITERIA METHOD=FISHER(1) SCALE=1 MAXITERATIONS=100 MAXSTEPHALVING=5 PCONVERGE=1E-006(ABSOLUTE)

SINGULAR=1E-012 ANALYSISTYPE=3(WALD) CILEVEL=95 LIKELIHOOD=FULL

/EMMEANS TABLES=Smoking_current SCALE=ORIGINAL

/REPEATED SUBJECT=StudyID WITHINSUBJECT=IncludedPregnancyNr SORT=YES CORRTYPE=EXCHANGEABLE

ADJUSTCORR=YES COVB=ROBUST MAXITERATIONS=100 PCONVERGE=1e-006(ABSOLUTE) UPDATECORR=1

/MISSING CLASSMISSING=EXCLUDE

/PRINT CPS DESCRIPTIVES MODELINFO FIT SUMMARY SOLUTION (EXPONENTIATED).

* Generalized Estimating Equations.

GENLIN Flare_postpartum (REFERENCE=FIRST) BY Pregnancy_IVF_ICSI_yesno (ORDER=DESCENDING)

/MODEL Pregnancy_IVF_ICSI_yesno INTERCEPT=YES

DISTRIBUTION=BINOMIAL LINK=LOGIT

/CRITERIA METHOD=FISHER(1) SCALE=1 MAXITERATIONS=100 MAXSTEPHALVING=5 PCONVERGE=1E-006(ABSOLUTE)

SINGULAR=1E-012 ANALYSISTYPE=3(WALD) CILEVEL=95 LIKELIHOOD=FULL

/EMMEANS TABLES=Pregnancy_IVF_ICSI_yesno SCALE=ORIGINAL

/REPEATED SUBJECT=StudyID WITHINSUBJECT=IncludedPregnancyNr SORT=YES CORRTYPE=EXCHANGEABLE

ADJUSTCORR=YES COVB=ROBUST MAXITERATIONS=100 PCONVERGE=1e-006(ABSOLUTE) UPDATECORR=1

/MISSING CLASSMISSING=EXCLUDE

/PRINT CPS DESCRIPTIVES MODELINFO FIT SUMMARY SOLUTION (EXPONENTIATED).

* Generalized Estimating Equations.

GENLIN Flare_postpartum (REFERENCE=FIRST) BY Flare_during Flare_last_in UC_yesno Previous_Surgery_yesno

Med_Biological_yesno Smoking_current Pregnancy_IVF_ICSI_yesno (ORDER=DESCENDING) WITH

DiseaseDuration

/MODEL Flare_during Flare_last_in UC_yesno DiseaseDuration Previous_Surgery_yesno Med_Biological_yesno

Smoking_current Pregnancy_IVF_ICSI_yesno INTERCEPT=YES

DISTRIBUTION=BINOMIAL LINK=LOGIT

/CRITERIA METHOD=FISHER(1) SCALE=1 MAXITERATIONS=100 MAXSTEPHALVING=5 PCONVERGE=1E-006(ABSOLUTE)

SINGULAR=1E-012 ANALYSISTYPE=3(WALD) CILEVEL=95 LIKELIHOOD=FULL

/EMMEANS TABLES=Flare_during SCALE=ORIGINAL

/EMMEANS TABLES=Flare_last_in SCALE=ORIGINAL

/EMMEANS TABLES=UC_yesno SCALE=ORIGINAL

/EMMEANS TABLES=Previous_Surgery_yesno SCALE=ORIGINAL

/EMMEANS TABLES=Med_Biological_yesno SCALE=ORIGINAL

/EMMEANS TABLES=Smoking_current SCALE=ORIGINAL

/EMMEANS TABLES=Pregnancy_IVF_ICSI_yesno SCALE=ORIGINAL

/REPEATED SUBJECT=StudyID WITHINSUBJECT=IncludedPregnancyNr SORT=YES CORRTYPE=EXCHANGEABLE

ADJUSTCORR=YES COVB=ROBUST MAXITERATIONS=100 PCONVERGE=1e-006(ABSOLUTE) UPDATECORR=1

/MISSING CLASSMISSING=EXCLUDE

/PRINT CPS DESCRIPTIVES MODELINFO FIT SUMMARY SOLUTION (EXPONENTIATED).
