# Supplementary material for: How long is long enough? Timing of pre-conceptional remission predicts relapse risk during pregnancy in IBD
Source: J Crohns Colitis. 2025 Oct 13;19(10):jjaf176. doi: 10.1093/ecco-jcc/jjaf176 (PMC12640223; doi:10.1093/ecco-jcc/jjaf176)
Supplement: jjaf176_Supplementary_Data [file jjaf176_supplementary_data.zip › Supplementary data 2 - Baseline differences cases versus controls.docx]

**Supplementary data 2**

**Baseline differences cases versus controls**

|  | **Cases and controls (*N*=884)** | **Cases (*N*=476)** | **Controls (*N*=408)** | ***p*-value** |
| --- | --- | --- | --- | --- |
| Phenotype (CD), *n* (%) | 587 (66.4) | 302 (63.4) | 285 (69.9) | **0.044** |
| Montreal classification, UC, *n* (%)  Proctitis  Leftsided  Extensive | 69 (23.2)  79 (26.6)  123 (41.4) | 53 (30.5)  44 (25.3)  58 (33.3) | 16 (13.0)  35 (28.5)  65 (52.9) | **<0.001**  0.724  **0.003** |
| Montreal classification, CD, *n* (%)  Ileum  Colon  Ileocolic  Upper GI  Perianal  Inflammatory  Stenosing  Fistulating | 201 (34.2)  86 (14.7)  265 (45.1)  68 (11.6)  124 (21.1)  385 (65.6)  106 (18.1)  57 (9.7) | 112 (37.1)  43 (14.2)  120 (39.7)  25 (8.3)  46 (15.2)  196 (64.9)  48 (15.9)  26 (8.6) | 89 (31.2)  43 (15.1)  145 (50.9)  43 (15.1)  78 (27.4)  189 (66.3)  58 (20.4)  31 (10.9) | **0.033**  0.957  **0.045**  **0.009**  **<0.001**  0.238  0.361  0.560 |
| Medication-use at conception, *n* (%)  Amino salicylates  Thiopurines  Corticosteroids  Budesonide  Anti-TNF  Vedolizumab  Ustekinumab  Certolizumab  Calcineurin inhibitors  Methotrexate | 681 (77.0)  208 (23.5)  253 (28.6)  20 (2.3)  20 (2.3)  282 (31.9)  54 (6.1)  44 (5.0)  5 (0.6)  9 (1.0)  20 (2.3) | 354 (74.4)  115 (24.2)  131 (27.5)  7 (1.5)  6 (1.3)  147 (30.9)  26 (5.5)  22 (4.6)  5 (1.1)  3 (0.6)  0 (0.0) | 327 (80.3)  93 (22.8)  122 (29.9)  13 (3.2)  14 (3.4)  135 (33.1)  28 (6.9)  22 (5.4)  0 (0.0)  6 (1.5)  20 (4.9) | **0.042**  0.633  0.435  0.087  **0.030**  0.483  0.386  0.600  **0.038**  0.215  **<0.001** |
| History of surgery, *n* (%) | 195 (22.1) | 93 (19.5) | 102 (25.0) | 0.051 |
| BMI at start of (dummy) pregnancy, median (IQR) | 24.3 (21.6 – 27.4) | 24.6 (22.0 – 28.2) | 24.0 (21.6 – 28.1) | 0.260 |
| Maternal age, median (IQR) | 31.8 (27.5 – 35.5) | 31.3 (28.0 – 34.2) | 31.5 (25.6 – 37.8) | 0.793 |
| Disease duration, median (IQR) | 6.9 (3.6 – 11.0) | 6.3 (3.1 – 10.1) | 8.3 (4.4 – 13.0) | **<0.001** |
| Currently smoking, *n* (%) | 132 (14.9) | 48 (10.1) | 84 (20.6) | **<0.001** |
