# Supplementary material for: How long is long enough? Timing of pre-conceptional remission predicts relapse risk during pregnancy in IBD
Source: J Crohns Colitis. 2025 Oct 13;19(10):jjaf176. doi: 10.1093/ecco-jcc/jjaf176 (PMC12640223; doi:10.1093/ecco-jcc/jjaf176)
Supplement: jjaf176_Supplementary_Data [file jjaf176_supplementary_data.zip › Supplementary data 3 - Complete outcome interaction analysis.docx]

**Supplementary data 3**

**Complete outcome interaction analysis**

|  | **Main effects, cases, univariable** | | | **Main effects, controls, univariable** | | | **Interaction** |
| --- | --- | --- | --- | --- | --- | --- | --- |
| *Characteristic* | OR | 95% CI | *p*-value | OR | 95% CI | *p*-value | *p*-value interaction term |
| *Last flare*  *- 0–3 months prior*  *- 3–6 months prior*  *- 6–12 months prior* | 5.018  2.897  1.616 | 2.6 – 9.9  1.1 – 8.0  0.8 – 3.1 | **<0.001**  **0.039**  0.152 | 5.372  3.769  3.612 | 2.8 – 10.4  2.0 – 7.1  1.9 – 6.9 | **<0.001**  **<0.001**  **<0.001** | 0.670 |
| *UC* | 0.934 | 0.6 – 1.4 | 0.757 | 0.554 | 0.3 – 0.9 | **0.017** | 0.188 |
| *Disease duration* | 0.984 | 1.0 – 1.0 | 0.434 | 0.990 | 1.0 – 1.0 | 0.546 | N.A. |
| *History of surgery* | 1.264 | 0.8 – 2.1 | 0.364 | 1.448 | 0.9 – 2.3 | 0.124 | N.A. |
| *Biological use* | 1.045 | 0.7 – 1.6 | 0.836 | 1.235 | 0.8 – 1.9 | 0.325 | N.A. |
| *Currently smoking* | 1.106 | 0.6 – 2.2 | 0.767 | 1.136 | 0.7 – 1.9 | 0.624 | N.A. |
|  | **Main effects, cases, multivariable** | | | **Main effects, controls, multivariable** | | | **Interaction** |
| *Characteristic* | aOR | 95% CI | *p*-value | aOR | 95% CI | *p*-value | *p*-value interaction term* |
| *Last flare*  *- 0–3 months prior*  *- 3–6 months prior*  *- 6–12 months prior* | 5.295  2.927  1.663 | 2.6 – 10.8  1.1 – 8.1  0.9 – 3.2 | **<0.001**  **0.040**  0.134 | 5.632  3.715  3.269 | 2.9 – 11.1  1.9 – 7.1  1.7 – 6.3 | **<0.001**  **<0.001**  **<0.001** | 0.736 |
| *UC* | 0.845 | 0.5 – 1.4 | 0.501 | 0.536 | 0.3 – 0.9 | **0.029** | 0.423 |
| *Disease duration* | 0.975 | 0.9 – 1.0 | 0.216 | 1.005 | 1.0 – 1.0 | 0.787 | N.A. |
| *History of surgery* | 1.141 | 0.7 – 2.0 | 0.635 | 1.038 | 0.6 – 1.8 | 0.893 | N.A. |
| *Biological use* | 0.910 | 0.6 – 1.4 | 0.667 | 0.926 | 0.6 – 1.5 | 0.740 | N.A. |
| *Currently smoking* | 1.214 | 0.6 – 2.4 | 0.582 | 0.974 | 0.6 – 1.7 | 0.925 | N.A. |

Abbreviations: aOR, adjusted odds ratio; CI, confidence interval; OR, odds ratio; UC, ulcerative colitis

*In the multivariable analysis, each interaction is assessed while accounting for the influence of the main effect of the other significant predictors. That is: The interaction term of pregnancy * last flare was corrected for the influence of phenotype, and the interaction term of pregnancy * phenotype was corrected for the influence of last flare.
